# Supplementary material for: Vital signs-based healthcare kiosks for screening chronic and infectious diseases: a systematic review
Source: Commun Med (Lond). 2025 Jan 21;5:28. doi: 10.1038/s43856-025-00738-5 (PMC11751283; doi:10.1038/s43856-025-00738-5)
Supplement: Supplementary file 2 — Supplementary Information [file 43856_2025_738_MOESM2_ESM.pdf]

Supplementary Information

Title: Vital signs-based Healthcare Kiosks for Screening Chronic and Infectious Diseases: A Systematic Review

Supplementary Note 1: Search Strategy

The PubMed, Institute of Electrical and Electronics Engineers (IEEE Xplore), and Google Scholar will be searched for articles published between Jan 1, 2013, and June 1, 2023. The detailed strategy on IEEE Xplore will be the following query: (("Full Text \& Metadata":kiosk OR "Full Text & Metadata":terminal OR "Full Text & Metadata":booth OR "Full Text & Metadata":platform) AND ("All Metadata":healthcare OR "All Metadata":hospital OR "All Metadata":clinic OR "All Metadata":nursing home OR "All Metadata":primary care) AND ("All Metadata":vital sign OR "All Metadata":physiological measurement OR "All Metadata":biometric OR "All Metadata":health parameter)). On Pubmed, the following search terms will be used: (kiosk OR terminal OR booth OR platform) AND (healthcare OR hospital OR clinic OR "nursing home" OR "primary care") AND (vital sign OR "physiological measurement" OR biometric OR "health parameter"). We are including Google Scholar into our study due to its reputation as a comprehensive database, particularly for grey literature sources. On Google Scholar the following terms will be used: (kiosk) AND (healthcare OR hospital OR clinic OR "nursing home" OR "primary care") AND ("vital sign" OR "physiological measurement" OR biometric OR "health parameter"). This timeframe was chosen to reflect advances in smart sensors, artificial intelligence technologies, and their kiosk applications in medicine.
