# Supplementary material for: Vital signs-based healthcare kiosks for screening chronic and infectious diseases: a systematic review
Source: Commun Med (Lond). 2025 Jan 21;5:28. doi: 10.1038/s43856-025-00738-5 (PMC11751283; doi:10.1038/s43856-025-00738-5)
Supplement: Supplementary file 3 — Description of Additional Supplementary Files [file 43856_2025_738_MOESM3_ESM.pdf]

Description of Additional Supplementary Files

File name: Supplementary Data 1

File description: a summary of key data extracted from the selected studies

File name: Supplementary Data 2

File description: Complete lists of all the 16/22 screened publications for this study are provided as an Excel file

File name: Supplementary Data 3

File description : presents the complete extracted data from all studies included in this review

File name: Supplementary Data 4

File description: Data about study limitation analysis

File name: Supplementary Data 5

File description: detailed assessment along with the adjudication process
