# Supplementary material for: Vital signs-based healthcare kiosks for screening chronic and infectious diseases: a systematic review
Source: Commun Med (Lond). 2025 Jan 21;5:28. doi: 10.1038/s43856-025-00738-5 (PMC11751283; doi:10.1038/s43856-025-00738-5)
Supplement: Supplementary file 6 — Supplementary Data 3 [file 43856_2025_738_MOESM6_ESM.pdf]

Title	Year	Study	First Author	Purpose	Vital Signs*	Contactless	No. of Sensors	Sensors	Gold Standard	Evaluation Metrics	Follows Standards?	Time of Use (seconds)	BP	HR	RR	Demographic	Number of Participants	Setting
Cost-Effective Solution of Remote Photoplethysmography Capable of Real-Time, Multi-Subject Monitoring with Social Distancing.	2022	Huang et al.	Huang	Screening for Cardiovascular Diseases	Heart Rate	Yes	1	RGB Camera	HR: No	N/R	N/R	N/R	N/A	rPPG	N/A	USA	11	Primary Care
Vitals: Camera-based Physiological Monitoring and Health Management Platform	2022	Wong et al.	Wong	Screening for Cardiovascular Diseases, Screening for Respiratory Diseases	Heart Rate, Blood Pressure, Blood Oxygen, Respiration Rate	Yes	1	RGB Camera	HR: No, BP: No, BO: No, RR: No	ACC_M 98/%	N/R	30 (avg)	rPPG	rPPG	rPPG	China	N/R	N/R
Contactless Diagnosis using Internet of Things (IoT) Technology for Covid-19 Suspect Patient	2022	Safi'ie et al.	Safi'ie	Screening for Infectious Diseases - Horizontal Transmission	Heart Rate, Body Temperature, Respiration Rate	No	3	Pulse Oximeter, IR Temperature, Air flow sensor	HR: No, BT: No, RR: No	ACC_S: 4.9 to 10.7\% (HR), Error: 1.6 to 4.3\% (RR), Error: 0.49 to 1.19 degree C (BT)	N/R	N/R	N/A	PPG	Air flow sensor	Indonesia	5	Community
Clinic, Home, and Kiosk Blood Pressure Measurements for Diagnosing Hypertension: a Randomized Diagnostic Study	2022	Green et al.	Green	Screening for Cardiovascular Diseases	Blood Pressure	No	2	Sphygmomanometer, Weighing Scale	BP: No	ACC_S [2.3 mmHg (Systolic) and 2.2 mmHg (Diastolic)]	N/R	N/R	N/A	N/A	N/A	USA	140	Primary Care
Hospital-based autonomous pre-clinical screening of COVID-19: An emergency triage using a vital signs recording system, Paris-Ile de France region.	2022	Brizio et al.	Brizio	Screening for Infectious Diseases - Horizontal Transmission	Heart Rate, Blood Pressure, Blood Oxygen, Body Temperature	No	5	Sphygmomanometer, Pulse Oximeter, IR Temperature, Weighing Scale, Height	HR: No, BO: No, BT: No	N/R	N/R	252 (avg)	N/A	N/A	N/A	France, USA	458	Secondary Care (ED)
Design and Development of Self-Service Telemedicine Kiosk for Remote Towns	2021	Vengadeshwaran et al.	Vengadeshwaran	Screening for Cardiovascular Diseases	Body Temperature, Heart Rate, Blood Pressure	No	2	IR Temperature, Pulse Oximeter	BT: No, HR: No	N/R	N/R	N/R	PPG	PPG	N/A	Malaysia	N/R	Community

Platform for Healthcare Promotion and Cardiovascular Disease Prevention	2021	Gómez et al.	Gómez	Screening for Cardiovascular Diseases	Blood Pressure, Blood Oxygen, Respiration Rate, Body Temperature	No	7	ECG, Pulse Oximeter, IR Temperature, Sphygmomanometer and Weighing Scale	BP: No, BO: No, RR: No, BT: No	ACC_M 83.9\% (Logistic Regression CVD classifier), ACC_M 82.8\% (Random Foreset CVD classifier)	N/R	N/R	N/A	N/A	ECG	Colombia	70	Community, Secondary Care (ED)
Automatic Health Machine for COVID-19 and Other Emergencies	2021	Ganesh et al.	Ganesh	Screening for Infectious Diseases - Horizontal Transmission	Body Temperature	No	3	PIR Sensor, IR Temperature Sensor and RGB Camera	BT: No	N/R	N/R	N/R	N/A	N/A	N/A	India	N/R	Community, Primary Care, Secondary Care (ED)
Can health kiosks be used to identify oral health care needs? A pilot study.	2020	Pentti et al.	Pentti	Screening for Dental Disease	N/A	No	5	RGB Cameras	N/A	N/R	N/R	371 (avg)	N/A	N/A	N/A	Finland	21	Secondary Care (Specality Clinic)
Designing Effective User Interface Experiences for a Self-Service Kiosk to Reduce Emergency Department Crowding	2020	Pacheco et al.	Pacheco	Screening for Cardiovascular Diseases	Blood Oxygen, Body Temperature, Blood Pressure	No	3	Pulse Oximeter, IR Temperature and Sphygmomanometer	BP: No, BO: No, BT: No	N/R	N/R	399 (avg)	N/A	N/A	N/A	Portugal	32	Secondary Care (ED)
Innovative product for premise safety from Covid 19: NeelKavach Kiosk	2020	Khetan et al.	Khetan	Screening for Infectious Diseases - Horizontal Transmission	Body Temperature	Yes	1	RGB Camera and Thermal Camera	BT: No	ACC_M 99\% (Mask Detection), ACC_S 0.3\degree C (Thermal Camera)	N/R	43 (avg)	N/A	N/A	N/A	India, UK	261	Community
Detecting Common Eye Diseases Using the First Teleophthalmology GlobeChek Kiosk in the United States: A Pilot Study	2020	Kapoor et al.	Kapoor	Screening for Eye disease	Blood Pressure	No	7	Sphygmomanometer, HbA1c, Optical Coherence Tomography Machine, ...	BP: No	$ACC_M 46.47\%$	N/R	N/R	N/A	N/A	N/A	USA	326	Community
A Novel Facial Thermal Feature Extraction Method for Non-Contact Healthcare System	2020	Wang et al.	Wang	Screening for Infectious Diseases - Horizontal Transmission	Body Temperature	Yes	2	RGB Camera and Thermal Camera	BT: No	ACC_M 85.96\% to 98\% (36-37.4 C)	N/R	N/R	N/A	N/A	N/A	Taiwan	10	N/R

AutoImpilo: Smart Automated Health Machine using IoT to Improve Telemedicine and Telehealth	2020	Ganesh et al.	Ganesh	Screening for Infectious Diseases - Horizontal Transmission, Screening for Cardiovascular Diseases	Heart Rate, Blood Pressure, Blood Oxygen, Body Temperature	No	3	Pulse Oximeter, IR Temperature and RGB camera	BP: No, BO: No, HR: No	N/R	N/R	N/R	N/R	N/R	N/A	India, UK	10	Community, Primary Care, Pharmacy
Blood Pressure Checks and Diagnosing Hypertension (BP-CHECK): Design and Methods of a Randomized Controlled Diagnostic Study Comparing Clinic, Home, Kiosk, and 24-Hour Ambulatory BP Monitoring	2019	Green et al.	Green	Screening for Cardiovascular Diseases	Blood Pressure	No	2	Sphygmomanometer, Weighing Scale	BP: No	ACC_S [4.1 mmHg (Systolic) and 2.8 mmHg (Diastolic)]	N/R	N/R	N/A	N/A	N/A	USA	136	Primary Care
Mixed-methods feasibility study of blood pressure self-screening for hypertension detection	2019	Tompson et al.	Tompson	Screening for Cardiovascular Diseases	Blood Pressure	No	1	Sphygmomanometer	BP: No	N/R	N/R	N/R	N/A	N/A	N/A	UK	186	Primary Care
Raspberry Pi-Based Medical Expert System for Pre-Diagnosis of Mosquito-Borne Diseases	2018	Magwili et al.	Magwili	Screening for Infectious Diseases - Vector Borne	Heart Rate, Blood Pressure, Body Temperature	No	3	Sphygmomanometer, IR Temperature, Pulse Oximeter	BP: No, BT: No, BO: No	ACC_M [71.67\% (Dengue), 83.33\% (Chikungunya), 91.67\% (Malaria)]	N/R	N/R	N/A	N/A	N/A	Philippines	80	Primary Care
A personal healthcare system for contact-less estimation of cardiovascular parameters	2018	Pasquadibisceglie et al.	Pasquadibisceglie	Screening for Cardiovascular Diseases	Heart Rate, Blood Oxygen	Yes	1	RGB Camera	HR: No, BO: No	ACC_S [2.87 BPM (HR) and 1.54% (BO)]	N/R	60 (avg)	N/A	rPPG	rPPG	Italy	25	Community
Contactless vital signs measurement for self-service healthcare kiosk in intelligent building	2018	Rizal et al.	Rizal	Screening for Cardiovascular Diseases, Screening for Respiratory Diseases	Blood Pressure, Respiration Rate (RR)	Yes	1	RGB Camera	RR: No, HR: No	ACC_S [1.7 BPM (PR), 0.41 BPM (RR) and 8.15 mmHg (SS)]	N/R	N/R	rPPG	N/A	rPPG	Taiwan	11	Community
IoT-based eHealth data acquisition system	2018	Pap et al.	Pap	Screening for Cardiovascular Diseases, Screening for Respiratory Diseases	Blood Pressure, Blood Oxygen, Body Temperature, Respiration Rate	No	5	Sphygmomanometer, Pulse Oximeter, IR Temperature, Galvanic Skin Response and Air Flow	BP: No, BO: No, RR: No, BT: No	N/R	N/R	N/R	N/A	N/A	Air flow sensor	Romania	N/R	N/R

Health outcomes of patients with chronic disease managed with a healthcare kiosk in primary care: protocol for a pilot randomised controlled trial	2018	Ng et al.	Ng	Screening for Cardiovascular Diseases, Screening for Metabolic Syndrome	Blood Pressure, Heart Rate	No	3	Sphygmomanometer, Height and Weighing Scale	BP: No, HR: No	N/R	No	N/R	N/R	N/R	N/A	Singapore	120	Primary Care
A Futuristic IOT Based Approach for Providing Healthcare Support through E-Diagnostic System in India	2017	Sarkar et al.	Sarkar	Screening for Cardiovascular Diseases	Heart Rate, Blood Pressure	No	5	Sphygmomanometer, Weighing Scale, Height, Digital Stethoscope and RGB Camera	HR: Yes, BP: No	N/R	N/R	360 to 420	N/A	N/A	N/A	India	N/R	Pharmacy
Usability assessment of a Health Kiosk	2017	Silva et al.	Silva	Screening for Cardiovascular Diseases	Blood Oxygen, Blood Pressure	No	4	Sphygmomanometer, Pulse Oximeter, Weighing Scale and RGB Camera	BP: No, BO: No	N/R	N/R	283 (avg)	N/A	N/A	N/A	Portugal	74	Community, Primary Care
Follow-Up Consultation Through a Healthcare Kiosk for Patients with Stable Chronic Disease in a Primary Care Setting: A Prospective Study	2017	Bahadin et al.	Bahadin	Screening for Cardiovascular Diseases, Screening for Metabolic Syndrome	Blood Pressure, Heart Rate	No	4	N/R	BP: No, HR: N/R	N/R	N/R	420 (avg)	N/R	N/R	N/A	Singapore	124	Primary Care
Multiple Vital-Sign-Based Infection Screening Outperforms Thermography Independent of the Classification Algorithm	2016	Yao et al.	Yao	Screening for Infectious Diseases - Horizontal Transmission	Heart Rate, Respiration Rate, Body Temperature	Yes	3	Thermal Camera, Respiration Radar, Laser Doppler Blood Flow	HR: No, RR: No, BT: No	N/R	N/R	34.9 [QDA] (avg)	N/A	N/A	N/A	Germany, Japan	92	N/R

Development of Automated Triage System for Emergency Medical Service	2016	Chong et al.	Chong	Screening for Cardiovascular Diseases, Screening for Respiratory Diseases, Screening for Metabolic Syndrome	Heart Rate, Blood Pressure, Blood Oxygen, Body Temperature, Respiration Rate	No	3	Sphygmomanometer, Pulse Oximeter, IR Temperature	HR: No, BP: No, BO: No, RR: No, BT: No	N/R	N/R	360	N/A	N/A	N/A	Malaysia	30	Secondary Care (ED)
Development of an Automated Healthcare Kiosk for the Management of Chronic Disease Patients in the Primary Care Setting	2016	Ng et al.	Ng	Screening for Cardiovascular Diseases, Screening for Metabolic Syndrome	Blood Pressure	No	3	Sphygmomanometer, Height and Weighing	BP: No	N/R	No	180 to 300	N/R	N/R	N/A	Singapore	100	Primary Care
Cloud based patient prioritization as service in public health care	2016	Bagula et al.	Bagula	Screening for Cardiovascular Diseases	Blood Oxygen, Body Temperature, Blood Pressure	No	4	Sphygmomanometer, Pulse Oximeter, IR Temperature	BP: No, BO: No, BT: No, HR: No	ACC_S 90.30\% (ML Model)	N/R	N/R	N/A	N/A	N/A	USA, Canada	2	Primary Care, Secondary Care (ED)
Designing and optimizing a healthcare kiosk for the community	2015	Lyu et al.	Lyu	Screening for Cardiovascular Diseases, Screening for Metabolic Syndrome	Blood Pressure, Blood Oxygen, Heart Rate	No	7	Sphygmomanometer, Pulse Oximeter, Glucometer, ECG, Weighing Scale and Height	BP: No, BO: No, HR: Yes	N/R	Yes	N/R	PPG	ECG	N/A	China, UK	32	N/R
Healthcare robot systems for a hospital environment: CareBot and ReceptionBot	2015	Ahn et al.	Ahn	Screening for Cardiovascular Diseases	Blood Pressure, Blood Oxygen	No	3	Sphygmomanometer, Pulse Oximeter	BP: No, BO: No	N/R	N/R	N/R	N/A	N/A	N/A	New Zealand	N/R	Primary Care
Comparison of an in-pharmacy automated blood pressure kiosk to daytime ambulatory blood pressure in hypertensive subjects	2015	Padwal et al.	Padwal	Screening for Cardiovascular Diseases	Blood Pressure	No	2	Sphygmomanometer, Weighing Scale	BP: No	ACC_S [5 mmHg (Systolic) and 3 mmHg (Diastolic)]	N/R	N/R	N/A	N/A	N/A	USA, Canada	100	Pharmacy

Design of a Kiosk Type Healthcare Robot System for Older People in Private and Public Places	2014	Ahn et al.	Ahn	Screening for Cardiovascular Diseases, Screening for Metabolic Syndrome	Blood Pressure, Blood Oxygen	No	4	Sphygmomanometer, Pulse Oximeter, RGB Camera, Glucometer	BP: No, BO: No	N/R	N/R	N/R	N/A	N/A	N/A	New Zealand	99	Community, Primary Care
Design of a healthcare sensor managing system for vital sign measuring devices	2014	Lee et al.	Lee	Screening for Cardiovascular Diseases	Blood Pressure, Blood Oxygen, Heart Rate	No	2	Sphygmomanometer, Pulse Oximeter	BP: No, BO: No, HR: No	N/R	N/R	N/R	N/A	N/A	N/A	New Zealand	N/R	N/R
ATM based Remote Healthcare Monitoring System	2014	Shibu et al.	Shibu	Screening for Cardiovascular Diseases, Screening for Metabolic Syndrome	Blood Pressure, Blood Oxygen, Body Temperature, Heart Rate	No	6	Sphygmomanometer, IR Temperature, Pulse Oximeter, Glucometer, Weight, Height	BP: No, BO: No, BT: No, HR: No	N/R	N/R	N/R	N/A	N/A	N/A	India	N/R	Primary Care
A smart phone/tablet based mobile health care system for developing countries	2013	Vaidya et al.	Vaidya	Screening for Cardiovascular Diseases	Blood Pressure, Heart Rate	No	3	Sphygmomanometer, Pulse Oximeter and ECG	BP: No, HR: Yes	N/R	N/R	N/R	PPG	ECG	N/A	India	N/R	Community
Consumer health information technology in an adult public health primary care clinic: a heart health education feasibility study	2013	Comstock et al.	Comstock	Screening for Cardiovascular Diseases	Blood Pressure	No	2	Sphygmomanometer and Weighing Scale	BP: No	N/R	N/R	N/R	N/A	N/A	N/A	USA	51	Primary Care
