# Supplementary material for: Vital signs-based healthcare kiosks for screening chronic and infectious diseases: a systematic review
Source: Commun Med (Lond). 2025 Jan 21;5:28. doi: 10.1038/s43856-025-00738-5 (PMC11751283; doi:10.1038/s43856-025-00738-5)
Supplement: Supplementary file 7 — Supplementary Data 4 [file 43856_2025_738_MOESM7_ESM.pdf]

Title	Study	Limitation in selection of participants	Limitation due to small sample size	Limitation due to non-response	Limitation due to selective result reporting	Limitation in measurement of outcome	Participants	Comments
Cost-Effective Solution of Remote Photoplethysmography Capable of Real-Time, Multi-Subject Monitoring with Social Distancing.	Huang et al. 2022	m	h	na	m	l	11	The study had 11 subjects, with diverse skin tones but between the ages of 20 to 30 years only. Two participants (3 and 7) were deemed outliers and were not considered.
Vitals: Camera-based Physiological Monitoring and Health Management Platform	Wong et al. 2022	na	na	na	h	l	N/R	The study mentions 98% accuracy on vital signs but the number of subject and criteria of selection of subjects is not mentioned.
Contactless Diagnosis using Internet of Things (IoT) Technology for Covid-19 Suspect Patient	Safi'ie et al. 2022	l	h	l	na	l	5	The study has only 5 participant of varied age groups (23 to 62 years). The error values for all the three sensors is below 10% for almost all the cases.
Clinic, Home, and Kiosk Blood Pressure Measurements for Diagnosing Hypertension: a Randomized Diagnostic Study	Green et al. 2022	l	l	l	l	l	140	This study compares the blood pressure measurements from kiosks, clinic, home with ABPM. 140 (48% female, 80% white, mean age 59 years) participants were included in the randomized control trial for kiosk measurements out of 28 were excluded due to complications with ABPM measurements. The error in measurements close to 2 mmHg.
Hospital-based autonomous pre-clinical screening of COVID-19: An emergency triage using a vital signs recording system, Paris-Ile de France region.	Brizio et al. 2022	l	l	h	l	na	458	This study uses kiosk for measuring vital signs for pre-clinical screening for Covid'19. The hospitals has 1844 patients visit during the study out of which only 458 (241 female, 217 male) choose to use the kiosk. The study does not report accuracy of measurement or diagnosis.
Design and Development of Self-Service Telemedicine Kiosk for Remote Towns	Vengadeshwaran et al. 2021	na	h	na	na	na	N/R	No information provided about testing.
Platform for Healthcare Promotion and Cardiovascular Disease Prevention	Gómez et al. 2021	h	m	l	l	h	70	The systems asks subjective questions along with measurements from the sensors as the input to machine learning models; 77% participants between 18 to 25; total error from sensors was close to 15%; error in classifiers is also close to 15%
Automatic Health Machine for COVID-19 and Other Emergencies	Ganesh et al. 2021	na	h	na	na	na	N/R	No information provided about testing.
Screening for Infectious Diseases	Pentti et al. 2020	h	h	h	l	l	21	Even though study only aims to analyse imaging technique on people in 20s, it only inlcludes 21 year olds in the study; only 21 particpants participate out of 192; study tests the results and determines negligible error in measurement
Designing Effective User Interface Experiences for a Self-Service Kiosk to Reduce Emergency Department Crowding	Pacheco et al. 2020	l	m	h	l	na	32	small sample size, though participants of various ages and educational levels were involved; Only 19 (41%) participants participated in the questionarre out of 32; error In measurement not specified
Innovative product for premise safety from Covid 19: NeelKavach Kiosk	Khetan et al. 2020	na	l	l	na	l	261	261 participants tested the kiosk but no information has been provided about the selection of the participants or the location where the test was conducted; no information has been provided about non-response, negligible error in sensor and ML model; study does not pre-define the data it is going to report
Detecting Common Eye Diseases Using the First Teleophthalmology GlobeChek Kiosk in the United States: A Pilot Study	Kapoor et al. 2020	l	l	m	l	h	326	71 out of 397 did not complete the screening, further 18 had unreadable images; thus total 22% participants did not complete or there data was not considered; the participants were diverse in the sense of age, sex and ethnicity; type 1 error is 0.05 and type 2 error is between 42.10% to 66.66%
A Novel Facial Thermal Feature Extraction Method for Non-Contact Healthcare System	Wang et al. 2020	na	h	na	na	l	10	The study had only 10 participants, and data from the same participants was used for training and testing. Participant selction has not been reported. The worst case error is 85.95% to 98%.
AutoImpilo: Smart Automated Health Machine using IoT to Improve Telemedicine and Telehealth	Ganesh et al. 2020	h	h	l	na	na	10	The study only had 10 participants, that too only 3 patients, 3 nurses and 4 doctors; only reveals the subjective view of participants about the kiosk; study does not pre-define the data it is going to report; study does not report non-response; error in measurements not reported
Blood Pressure Checks and Diagnosing Hypertension (BP- CHECK): Design and Methods of a Randomized Controlled Diagnostic Study Comparing Clinic, Home, Kiosk, and 24-Hour Ambulatory BP Monitoring	Green et al. 2019	l	l	l	l	l	136	This study compares the blood pressure measurements from kiosks, clinic, home with ABPM. 140 (48% female, 80% white, mean age 59 years) participants were included in the randomized control trial for kiosk measurements out of 28 were excluded due to complications with ABPM measurements. The error in measurements close to 2 mmHg.
Mixed-methods feasibility study of blood pressure self-screening for hypertension detection	Tompson et al. 2019	m	l	h	l	na	186	Only 1.2% of the eligible participants at used the kiosk; participants were sufficiently varied in gender but not as much in age (36 to 69); study reported all the results pre-defined in the methodology; error in measurements not reported
Raspberry Pi-Based Medical Expert System for Pre-Diagnosis of Mosquito-Borne Diseases	Magwili et al. 2018	m	m	l	l	h	80	The study has 80 participants, but 75% of them are diagnosed with mosquito borne disease and only 25% are without diseases. The accuracy of disease prediction system varies from 71% to 90%.
A personal healthcare system for contact-less estimation of cardiovascular parameters	Pasquadibisceglie et al. 2018	l	m	l	l	l	25	The study has 25 subjects, out of which 19 are males and 6 are females. Furthermore, the age of the subjects varies between 18 to 65 years and people of varying skin colour are present.
Contactless vital signs measurement for self-service healthcare kiosk in intelligent building	Rizal et al. 2018	m	h	l	na	m	11	Only 11 participants, though diversity in skin tone was ensure by fitzpatrick test, no diversity in genger (10:1) ang age (only 23 to 30 year olds) was present; study does not pre-define the data it is going to report; considerable MEA was observed
IoT-based eHealth data acquisition system	Pap et al. 2018	na	h	na	na	na	N/R	No information provided about testing.

Health outcomes of patients with chronic disease managed with a healthcare kiosk in primary care: protocol for a pilot randomised controlled trial	Ng et al. 2018	na	h	na	h	na	120	Prospective Study; the recruitment is planned to be randomized and later stratified; 120 participants are expected to be recruited; though the paper just outlines how the study will be performed, but does not actally perform it; none of the pre-defined results is provided
A Futuristic IOT Based Approach for Providing Healthcare Support through E-Diagnostic System in India	Sarkar et al. 2017	na	h	na	na	na		No information provided about testing.
Usability assessment of a Health Kiosk	Silva et al. 2017	m	m	l	l	na	74	74 participants who were students, teachers, or non teaching staff; error in measurement is not reported; study does not pre-define the data it is going to report
Follow-Up Consultation Through a Healthcare Kiosk for Patients with Stable Chronic Disease in a Primary Care Setting: A Prospective Study	Bahadin et al. 2017	l	l	h	l	l	124	selection of participants as per the primare outcome were diverse in terms of age, comorbidities, gender and ethnicity; initially for 17 participants the algorithm was incorrect and there data had to be discarded; further 36 participants quit over the period; strong agreement was found between the kiosk algorithm and medical staff; the study reports all the pre-defined data in the methodology
Multiple Vital-Sign-Based Infection Screening Outperforms Thermography Independent of the Classification Algorithm	Yao et al. 2016	l	m	l	l	m	92	The study had 92 participants, 57 (49 male and 8 female, 19–40 years) of which were diagnosed to have influenza and 35 (30 male and 5 female, 20–35 years) without influenza. The best performing method QDA was reported to have an error rate of 9.8%.
Development of Automated Triage System for Emergency Medical Service	Chong et al. 2016	na	m	l	l	na	30	To evaluate the system the authors choose 30 real case samples obtained from UKMMC and test on this. No information has been provided on the selction of these cases and the diversity of patients these cases had.
Development of an Automated Healthcare Kiosk for the Management of Chronic Disease Patients in the Primary Care Setting	Ng et al. 2016	m	m	l	m	m	100	70% participants were between 61 and 70 years of age, though diverse in gender and ethnicity; 4 withdrew; all the reading were double checked by nurse and faulty readings were not considered; 39% of error in decision of the algorithm was found during the first month; error for the first month of the study is mentioned but subsequent error is not mentioned, it is only mentioned that the results were accurate
Cloud based patient prioritization as service in public health care	Bagula et al. 2016	h	h	l	na	m	2	tested on 2 healthy participants; medically approved sensors used, moderate error is seen in the ML model
Designing and optimizing a healthcare kiosk for the community	Lyu et al. 2015	h	m	l	l	l	32	37.5% femaies, mean age 27.2; no significant error in measurement observed; the study reports all the pre-defined data in the methodology
Healthcare robot systems for a hospital environment: CareBot and ReceptionBot	Ahn et al. 2015	na	h	na	na	na	N/R	No information provided about testing.
Comparison of an in-pharmacy automated blood pressure kiosk to daytime ambulatory blood pressure in hypertensive subjects	Padwal et al. 2015	l	m	l	l	l	100	111 participants were screened out of which 9 dropped out and 2 were excluded. The participants were diverse with 53% females, average age of 59.7, 41% had a history of type 2 diabetes, and 6% had prior cerebro- vascular disease.
Design of a Kiosk Type Healthcare Robot System for Older People in Private and Public Places	Ahn et al. 2014	m	m	na	l	na	99	The robot kiosk platform is used by 99 older adult in private appartments, rest homes and hospitals in a 12 week long study. The study only reports the results on usability of the kiosk.
Design of a healthcare sensor managing system for vital sign measuring devices	Lee et al. 2014	na	h	na	na	na	N/R	Only information in terms of the latency of the sensors is provided. No information provided about testing.
ATM based Remote Healthcare Monitoring System	Shibu et al. 2014	na	h	na	na	na	N/R	No information provided about testing.
A smart phone/tablet based mobile health care system for developing countries	Vaidya et al. 2013	na	na	na	na	na	N/R	Study mentions about testing, no results of the test are mentioned
Consumer health information technology in an adult public health primary care clinic: a heart health education feasibility study	Comstock et al. 2013	h	m	h	l	l	51	Participants with BMI >=25 were only recruited, 84% female, and 96% african american; only 52% participants completed all three sessions; the study reports all the pre-defined data in the methodology; minimal error in observation was observed

Low	10	7	16	18	13
Moderate	7	12	1	2	4
High	6	15	6	2	3
Total determined	23	34	23	22	20
Total not determined	13	2	13	14	16
% Low	44	21	70	82	65
% Moderate	30	35	4	9	20
% High	26	44	26	9	15

	(red)	(orange)	(green)
Number of participants	<25	>=25 to <120	>=120
Error in measurement/algorithm	>=10%	>=5% to <10%	<=5%`
Percetage of participants that do not complete the study or part of the study	>=30%	>=10% to <30%	<10%

Limitation Level

Low	(green)
Moderate	(orange)
High	(red)
Not Applicable	(grey)
