# Supplementary material for: Vital signs-based healthcare kiosks for screening chronic and infectious diseases: a systematic review
Source: Commun Med (Lond). 2025 Jan 21;5:28. doi: 10.1038/s43856-025-00738-5 (PMC11751283; doi:10.1038/s43856-025-00738-5)
Supplement: Supplementary file 8 — Supplementary Data 5 [file 43856_2025_738_MOESM8_ESM.pdf]

Study	Reviewer 1 (S.B.)					Reviewer 2 (M.E.)					Adjudicated				
	Limitation in selection of participants	Limitation due to small sample size	Limitation due to non-response	Limitation due to selective result reporting	Limitation in measurement of outcome	Limitation in selection of participants	Limitation due to small sample size	Limitation due to non-response	Limitation due to selective result reporting	Limitation in measurement of outcome	Limitation in selection of participants	Limitation due to small sample size	Limitation due to non-response	Limitation due to selective result reporting	Limitation in measurement of outcome
Huang et al. 2022	m	h	na	na	l	m	h	na	m	l	m	h	na	m	l
Wong et al. 2022	na	na	na	h	l	na	na	na	m	l	na	na	na	h	l
Safi'ie et al. 2022	l	h	l	na	m	l	h	l	na	l	l	h	l	na	l
Green et al. 2022	l	l	l	m	l	m	l	l	l	l	l	l	l	l	l
Brizio et al. 2022	l	l	h	l	na	l	l	h	l	na	l	l	h	l	na
Vengadeshwaran et al. 2021	na	na	na	na	h	na	h	na	na	na	na	h	na	na	na
Gómez et al. 2021	h	m	l	l	m	h	m	l	l	h	h	m	l	l	h
Ganesh et al. 2021	na	na	na	na	h	na	h	na	na	na	na	h	na	na	na
Pentti et al. 2020	h	h	h	l	l	h	h	h	l	l	h	h	h	l	l
Pacheco et al. 2020	l	m	m	l	l	l	m	h	l	na	l	m	h	l	na
Khetan et al. 2020	na	l	l	m	l	na	l	l	na	l	na	l	l	na	l
Kapoor et al. 2020	l	l	m	l	l	l	l	m	l	h	l	l	m	l	h
Wang et al. 2020	na	h	na	na	l	na	h	na	na	na	na	h	na	na	l
Ganesh et al. 2020	h	h	l	na	na	h	h	l	na	na	h	h	l	na	na
Green et al. 2019	l	l	l	l	l	m	l	l	l	l	l	l	l	l	l
Tompson et al. 2019	m	l	h	l	na	m	l	h	l	na	m	l	h	l	na
Magwili et al. 2018	m	m	l	l	h	h	m	l	l	h	m	m	l	l	h
Pasquadibisceglie et al. 2018	l	m	l	l	l	l	m	l	l	l	l	m	l	l	l
Rizal et al. 2018	m	h	l	na	m	m	h	l	na	m	m	h	l	na	m
Pap et al. 2018	na	h	na	na	na	na	h	na	na	na	na	h	na	na	na
Ng et al. 2018	na	na	na	l	na	na	h	na	h	na	na	h	na	h	na
Kwok et al. 2018	l	m	l	l	na	l	m	l	l	na	l	m	l	l	na
Sarkar et al. 2017	na	h	na	na	na	na	h	na	na	na	na	h	na	na	na
Silva et al. 2017	m	m	l	l	na	m	m	l	l	na	m	m	l	l	na
Bahadin et al. 2017	l	l	h	l	l	l	l	h	l	l	l	l	h	l	l
Yao et al. 2016	m	m	l	l	m	l	m	l	l	m	l	m	l	l	m
Chong et al. 2016	na	m	l	l	na	na	m	l	l	na	na	m	l	l	na
Ng et al. 2016	m	m	l	l	l	m	m	l	m	m	m	m	l	m	m
Bagula et al. 2016	h	h	l	na	l	h	h	l	na	m	h	h	l	na	m
Lyu et al. 2015	m	m	l	l	l	h	m	l	l	l	h	m	l	l	l
Ahn et al. 2015	na	na	na	na	na	na	h	na	na	na	na	h	na	na	na
Padwal et al. 2015	m	m	l	l	l	l	m	l	l	l	l	m	l	l	l
Ahn et al. 2014	m	h	na	l	na	m	m	na	l	na	m	m	na	l	na
Lee et al. 2014	na	h	na	na	na	na	h	na	na	na	na	h	na	na	na
Shibu et al. 2014	na	h	na	na	na	na	h	na	na	na	na	h	na	na	na
Vaidya et al. 2013	na	na	na	na	na	na	na	na	na	na	na	na	na	na	na
Comstock et al. 2013	m	m	h	l	l	h	m	h	l	l	h	m	h	l	l

	Low	Moderate	High	NA
Low	57	5	2	4
Moderate	4	24	4	1
High	0	2	25	2
NA	0	1	4	70

Cohen's Kappa	0.802
